# Supplementary material for: Comparative multiomics analysis of cell physiological state after culture in a basket bioreactor
Source: Sci Rep. 2022 Nov 23;12:20161. doi: 10.1038/s41598-022-24687-4 (PMC9686226; doi:10.1038/s41598-022-24687-4)
Supplement: Supplementary file 1 — Supplementary Information 1. [file 41598_2022_24687_MOESM1_ESM.zip › raw data/Transcriptomic raw data/6.Enrichment/2.KEGG/TE1vsTC1/ALL/src/csab05206.html]

csab05206


- csab:103247197

- Up regulated genes

103247197(1.34087353024886)

- csab:103246489

- Up regulated genes

103246489(1.48197213715262)

- csab:103214617

- Up regulated genes

103214617(2.5646577284272)

- csab:103237430

- Up regulated genes

103237430(1.35562492669487)

- csab:103224900

- Down regulated genes

103224900(-1.42998371610984)

- csab:103214617

- Up regulated genes

103214617(2.5646577284272)

- csab:103214788

- Up regulated genes

103214788(1.09310575924899)

- csab:103239621

- Down regulated genes

103239621(-1.32776691017615)

- csab:103246489

- Up regulated genes

103246489(1.48197213715262)

- csab:103243277

- Up regulated genes

103243277(1.88743299439371)

- csab:103239621

- Down regulated genes

103239621(-1.32776691017615)

- csab:103246479

- Down regulated genes

103246479(-1.5490263767958)

- csab:103246479

- Down regulated genes

103246479(-1.5490263767958)

- csab:103246479

- Down regulated genes

103246479(-1.5490263767958)

- csab:103246479

- Down regulated genes

103246479(-1.5490263767958)

Close
